# Improving mental health care in humanitarian emergencies

**DOI:** 10.2471/BLT.15.156919

**Published:** 2015-10-01

**Authors:** Peter Ventevogel, Mark van Ommeren, Marian Schilperoord, Shekhar Saxena

**Affiliations:** a Public Health Section, Division of Programme Management and Support, United Nations High Commissioner for Refugees, 94 Rue de Montbrillant, 1202 Geneva, Switzerland.; b Department of Mental Health and Substance Abuse, World Health Organization, Geneva, Switzerland.

The mental health needs of people affected by emergencies are significant, but often overlooked by health-care providers.^1^ The world is facing an unprecedented number of humanitarian emergencies arising from conflict and disasters. In 2014, nearly 60 million people were forcibly displaced due to conflict, the highest number on record.^2^ Climatic and geological hazards continue to take their toll, as seen recently following the devastating earthquake in Nepal, cyclone in Vanuatu and flooding in China, Malawi and Myanmar.

Although estimated rates of mental disorder after conflict vary due to differences in context and study methods, a meta-analysis of methodically stronger surveys indicate average rates of 15–20% for depression and post-traumatic stress disorder.^3^ This is in line with projected rates of mental disorder after disasters.^4^ In humanitarian emergencies, mental health complaints are diverse in nature and severity.

First, grief and acute stress are usually transient psychological reactions to adversity and loss. These require a basic, supportive psychosocial response.^5^ Yet, when these reactions interfere with daily functioning – as is the case when people develop associated symptoms of insomnia, enuresis or hyperventilation – general health-care providers (e.g. non-specialized physicians, clinical officers and nurses) need to know how to manage this.

Second, common mental disorders, such as depression, post-traumatic stress disorder, and prolonged grief disorder, may be triggered by extreme stressors (e.g. trauma and loss). These disorders may become chronic and undermine the functioning of individuals and communities, which is essential for their survival and socioeconomic recovery. Health-care providers need to know how to manage these problems and how to distinguish mental disorders from widespread emotional distress that is common in emergency settings.^6^

Third, pre-existing chronic psychoses, bipolar disorder, intellectual disability and epilepsy can cause significant vulnerability in the chaos of an emergency. People with these conditions are at particular risk of neglect, abandonment, abuse, interruption of maintenance medication and lack of access to health services. Moreover, triggered by the stress of adversity, people with a history of severe mental disorder may experience a relapse or exacerbation of existing symptoms.^7^

Finally, acute health risks and social problems due to alcohol and drug use can be magnified in humanitarian settings;^8^ health-care providers need to be able to manage harmful use of alcohol and drugs as well as life-threatening withdrawal.^5^

There is consensus that humanitarian assistance should address mental health and psychosocial issues through intersectoral action.^5^^,^^9^ Currently, most health agencies do not routinely address these needs, though the programmes of Médecins Sans Frontières and the International Medical Corps are notable exceptions. Many international humanitarian organizations initiate important community-based psychosocial support interventions outside the health sector (e.g. child-friendly spaces, linking vulnerable people to resources) but ignore clinical intervention through health services.^10^ A recent analysis of records from 90 refugee camps confirms that mental health care is rarely provided: the average consultation rate across all camps for mental, neurological and substance use conditions was 4.3 visits per 1000 persons per month,^11^ while the estimated prevalence rate of these conditions is much higher.^4^

To address these gaps in service provision, the World Health Organization and the United Nations High Commissioner for Refugees have released the *mhGAP Humanitarian Intervention Guide*.^12^ This practical tool will help enable health-care providers in assessing and offering first-line management of mental, neurological and substance use conditions in humanitarian emergencies. The new guide is adapted from the *mhGAP Intervention Guide*^13^ a widely-used evidence-based manual for the management of these conditions.

During humanitarian crises, health systems tend to be overwhelmed and unable to meet the demand for basic services. Often, existing supportive care systems in the communities have been damaged. Human resources tend to be overstretched, with limited time for training. Access to specialists for referral and support is typically limited, while medication supply lines may be disrupted. Therefore, it is important to determine how interventions with proven efficacy can be most effectively scaled up in emergencies and refugee settings.^14^ We call upon all humanitarian health actors to implement agreed policy on mental health care^15^ and routinely include and evaluate clinical mental health care in their basic package of primary health services.^12^ This will help to reduce mental suffering and improve the well-being and functioning of people affected by armed conflicts and disasters.^16^
